# CD25^+^CD27^+^CD70^−^ alloantigen-specific Tregs: promising stable immunotherapy for transplantation

**DOI:** 10.3389/fimmu.2026.1789008

**Published:** 2026-06-10

**Authors:** Arimelek Cortés-Hernández, Saúl Arteaga-Cruz, Iyari I. Martínez Iturbe, Katya Rosas-Cortina, Marco A. Vigil Mora, Erick Legorreta-Anguiano, Judith E. Reyes Barrientos, Evelyn K. Álvarez-Salazar, Alejandra Cervera, Beatriz E. Sánchez-Hernández, Armando Gamboa Domínguez, Gloria Soldevila

**Affiliations:** 1 Department of Immunology, Biomedical Research Institute, National Autonomous University of Mexico, Mexico City, Mexico; 2 National Laboratory of Flow Cytometry, Biomedical Research Institute, National Autonomous University of Mexico, Mexico City, Mexico; 3 Biomedical Genomics and Bioinformatics Laboratory, Instituto Nacional de Medicina Genómica (INMEGEN), Mexico City, Mexico; 4 Department of Pathology, Instituto Nacional de Ciencias Médicas y la Nutrición Salvador Zubirán, Mexico City, Mexico

**Keywords:** adoptive cell therapy, alloantigen-specific Tregs, CD27, lineage stability, transplantation

## Abstract

The clinical translation of alloantigen-specific regulatory T cells (AS-Tregs) is constrained by their low frequency in peripheral blood, limited purity, and functional instability following prolonged *ex vivo* expansion. To address these hurdles, we developed a strategy to isolate and expand a functional CD25^+^CD27^+^CD70^−^ AS-Treg population. Initially, Tregs were co-cultured with allogeneic dendritic cells in the presence of IL-15, IL-2, and retinoic acid. Proliferating CD25^+^CD27^+^CD70^−^ AS-Tregs were subsequently FACS-sorted and expanded via polyclonal anti-CD3/CD28 stimulation with IL-15, IL-2, rapamycin, and TGF-β. Over three weeks, this protocol yielded a 434-fold expansion, achieving >95% purity (CD25^+^FOXP3^+^) while maintaining a substantial degree (>60%) of *FOXP3*-TSDR demethylation, a hallmark of stable Treg lineage commitment. The expanded CD27^+^ AS-Tregs exhibited a robust immunoregulatory phenotype, characterized by high expression of Helios, CTLA-4, and CD39, as well as chemokine receptors associated with allograft and lymphoid tissue homing (CXCR3, CCR4, and CCR7). Functionally, CD27^+^ AS-Tregs suppressed T cell proliferation in an antigen-specific manner, even after exposure to inflammatory cytokines, and showed a concentration-dependent chemotactic response to CXCL10 *in vitro*. In addition, these cells maintained lineage fidelity by lacking the production of inflammatory cytokines such as IFN-γ and IL-17A. Accordingly, transcriptional profiling by RNA sequencing confirmed the enrichment of immunoregulatory signatures and revealed minimal changes in gene expression when expanded Tregs were exposed to inflammatory conditions. Overall, our findings suggest that CD27^+^ AS-Tregs represent promising candidates for more stable, long-term Treg therapy to support transplant tolerance.

## 1 Introduction

Regulatory T cells (Tregs) play a pivotal role in establishing and maintaining allograft tolerance by suppressing conventional T cells and modulating the activity of antigen-presenting cells (1). Treg function critically relies on the transcription factor FOXP3, which governs their immunosuppressive program (2). In humans, liver and kidney transplant recipients who achieve clinical operational tolerance exhibit a significant increase in circulating FOXP3^+^ Tregs (3, 4). This observation has inspired Phase I and Phase I/IIa clinical trials utilizing polyclonally expanded Tregs, which have demonstrated robust safety profiles without increasing infection or rejection risks during follow-up (5–8). However, preclinical data consistently indicate that alloantigen-specific Tregs (AS-Tregs) possess superior efficacy for inducing long-term allograft tolerance compared to their polyclonal counterparts (9–12). Consequently, translating AS-Tregs into clinical practice represents a major goal in transplantation, offering the promise of durable immune regulation with reduced reliance on systemic immunosuppression.

Despite their clear therapeutic potential, the large-scale manufacturing of human AS-Tregs is significantly constrained by their low precursor frequency in peripheral blood (13, 14). While engineering Tregs with chimeric antigen receptors (CARs) or transgenic T-cell receptors (TCRs) has emerged as a strategy to bypass this numerical limitation, these approaches entail high costs, complex genetic manipulation, and unique regulatory hurdles (14–16). Furthermore, achieving the necessary cell yields for natural AS-Tregs requires prolonged *ex vivo* expansion, which inherently carries the risk of FOXP3 downregulation and pathogenic conversion into pro-inflammatory effector cells, potentially compromising their clinical applicability (17, 18).

In a previous study, we addressed the challenge of *ex vivo* expansion by validating a protocol using allogeneic monocyte-derived dendritic cells to drive the specific proliferation of AS-Tregs (19). While that protocol yielded functional FOXP3^+^ cells, unexpectedly the isolation of AS-Tregs based solely on CD25 expression led to a significant decrease in *FOXP3*-locus demethylation after expansion, a factor known to compromise lineage stability *in vivo* (20). This observation indicates that refining selection markers is necessary to ensure the phenotypic homogeneity and lineage stability required for clinical applications. In this context, recent evidence demonstrates that the CD27^+^CD70^−^ phenotype defines a Treg subset with preserved suppressive capacity and epigenetic stability (21). Furthermore, alongside lineage stability, the therapeutic efficacy of infused Tregs relies on their capacity to home to both lymphoid tissues and the graft site via the expression of specific chemokine receptors (22).

Building on these insights, the aim of this study was to develop a robust strategy for the isolation and sustained expansion of a high functional CD25^+^CD27^+^CD70^−^ AS-Treg population. By integrating alloantigen-driven proliferation with CD27^+^ selection and CD70^−^ exclusion, our pipeline overcomes the epigenetic instability typical of long-term AS-Treg cultures. We show that these expanded cells retain their regulatory identity and exert robust, antigen-specific suppression, while co-expressing the essential chemokine receptors required for allograft and lymphoid homing. Importantly, this functional resilience was associated with sustained *FOXP3*-TSDR demethylation and the maintenance of a resilient regulatory transcriptomic profile, even under inflammatory conditions.

## 2 Materials and methods

### 2.1 Reagents and antibodies

For flow cytometry, APC-Cy7 anti-CD4, APC anti-CD3, PE-Cy7 anti-CD27, PE-Cy7 anti-CD8, and the FOXP3/Transcription Factor Staining Buffer Kit were procured from Tonbo Biosciences (San Diego, CA, USA). Brilliant Violet 605 anti-CD4, PE-CF594 anti-CD70, Alexa Fluor 647 anti-FOXP3, Alexa Fluor 488 anti-IL-17A, and PE-Cy7 anti-IFN-γ were sourced from BD Biosciences (Franklin Lakes, USA). APC-Fire750 anti-CD73, APC-Fire750 anti-CD45RA, Brilliant Violet 711 anti-CD39, Brilliant Violet 421 anti-CTLA-4, FITC anti-Helios, PE anti-CD25, PE-Cy7 anti-CD127, Brilliant Violet 421 anti-CCR4, Brilliant Violet 711 anti-CXCR3, Brilliant Violet 605 anti-CCR2, PerCP-Cy5.5 anti-CCR7, and Zombie Aqua™ fixable viability dye were supplied by BioLegend (San Diego, USA).

For *in vitro* experiments, rapamycin, all-trans-retinoic acid (ATRA), Ficoll^®^ Paque Plus, and dimethyl sulfoxide (DMSO) were obtained from Sigma-Aldrich (Saint Louis, USA). Recombinant human cytokines CXCL10, GM-CSF, IFN-γ, IL-2, IL-4, IL-6, IL-15, TGF-β, and TNF-α were purchased from PeproTech (Cranbury, USA). CFSE CellTrace™ (CFSE), CellTrace™ Violet (CTV) and CellTrace Yellow™ (CTY) proliferation kits, Dynabeads™ Human T-Activator CD3/CD28 (anti-CD3/CD28 beads), DynaMag-5™ Magnet, CTS™ OpTmizer™ T Cell Expansion SFM (TCE medium), RPMI 1640 medium, antibiotic–antimycotic (100×), GlutaMAX™ (100×), sodium pyruvate (100 mM), MEM non-essential amino acids (100×), fetal bovine serum (FBS), and PureLink™ RNA Mini Kit were purchased from Thermo Fisher Scientific (Waltham, USA). Pooled human AB serum was procured from Gemini Bio Products (Sacramento, USA). All media were supplemented with GlutaMAX™, sodium pyruvate, MEM-NEAA, and antibiotic–antimycotic. T cell cultures were performed in 96-well round-bottom plates (Corning, Avon, France).

### 2.2 Study population and sample collection

Buffy coat preparations were obtained from the Blood Bank of the National Institute of Medical Sciences and Nutrition Salvador Zubirán (INCMNSZ), utilizing samples from thirteen healthy donors aged 18 to 55 years. Inclusion criteria required the absence of neoplastic disease, acute or chronic inflammatory disorders, or active infectious processes. The studies were conducted in accordance with the local legislation and institutional requirements.

### 2.3 PBMC Isolation and Pre-screening of alloreactive mismatched pairs

Peripheral blood mononuclear cells (PBMCs) were isolated from healthy donors by density-gradient centrifugation using Ficoll-Paque™ PLUS, following the manufacturer’s instructions. For cryopreservation, PBMCs were resuspended in a cold freezing medium (90% FBS, 10% DMSO), frozen at −70 °C for 24 h, and subsequently transferred to liquid nitrogen for long-term storage. Prior to functional assays, cells were rapidly thawed at 37 °C, washed with RPMI 1640 medium supplemented with 10% FBS, and resuspended in culture medium.

Optimal mismatched donor pairs for Treg expansion were selected based on baseline alloreactivity. This was performed via Mixed Lymphocyte Reactions (MLRs) using PBMCs labeled with CellTrace™ dyes (Thermo Fisher Scientific) according to the manufacturer’s protocol. Donor pairs demonstrating >10% of allo-proliferation were subsequently chosen for the specific expansion protocols.

### 2.4 Generation of monocyte-derived dendritic cells

CD14^+^ monocytes were purified from PBMCs using the Human CD14 MicroBeads Kit (Miltenyi Biotec, Germany), according to the manufacturer’s protocol. Purified monocytes were cultured in RPMI 1640 supplemented with 10% human AB serum and stimulated with IL-4 (50 ng/mL) and GM-CSF (50 ng/mL) for 8 days. On days 3 and 5, a partial medium exchange was performed, and cultures were supplemented with IL-4 (25 ng/mL) and GM-CSF (25 ng/mL). Subsequently, Mo-DCs were washed with culture medium and irradiated at 3000 rads before use in culture assays. For functional assays, Mo-DCs were further matured on day 5 using the pro-inflammatory cytokines IL-6, IL-1β, and TNF-α at a concentration of 10 ng/mL.

### 2.5 Isolation and expansion of allospecific Tregs

Treg and Tconv Isolation: PBMCs were stained with anti-CD4, anti-CD25, anti-CD127 and anti-CD45RA monoclonal antibodies, washed, and resuspended in phosphate-buffered saline (PBS). The CD4^+^CD25^+^CD127^−^ and CD4^+^CD25^−^CD45RA^+^ populations were sorted as Tregs and naïve conventional T cells, respectively, using a FACSAria Fusion cell sorter (BD Biosciences, USA).

Alloantigen and polyclonal primary stimulations: Isolated CD4^+^CD25^+^CD127^−^ Tregs were labeled with CTV following the manufacturer’s protocol. Cells (5×10^5^ cells/well) were stimulated with either irradiated allogeneic MoDCs (to generate allospecific Tregs, AS-Tregs) or anti-CD3/CD28 beads (to generate polyclonal Tregs, Poly-Tregs). Both conditions were established at a 2:1 Treg-to-stimulator ratio. Cultures were maintained in TCE medium supplemented with 10% human AB serum, ATRA (10 nM), IL-15 (10 ng/mL), and IL-2 (250 U/mL).

Sorting of allospecific and control Tregs: On day 7 of culture, cells from both allogeneic and polyclonal expansions were stained with anti-CD4, anti-CD25, anti-CD27, anti-CD70, and a viability dye. From the allogeneic co-cultures, three distinct CD4^+^CD25^+^ subpopulations were isolated by FACS based on their proliferation status and CD27/CD70 expression: the actively dividing (CTV^low^) CD27^+^CD70^−^ fraction (defined as CD27^+^ AS-Tregs), the non-proliferating (CTV^+^) CD27^+^CD70^−^ fraction (defined as CD27^+^ Non-AS Tregs), and the dividing (CTV^low^) CD27^−^CD70^+^ fraction (defined as CD70^+^ AS-Tregs), the latter serving as an additional control (**Supplementary Figure 1**). In parallel, the actively dividing (CTV^low^) CD4^+^CD25^+^CD27^+^CD70^−^ cells from polyclonal cultures were sorted to generate the CD27^+^ Poly-Treg group. All sorted populations were collected in RPMI medium supplemented with FBS, and subsequently cultured for 3 days in TCE medium supplemented with IL-2 (50 U/mL) and 10% human AB serum to rest.

Polyclonal Expansion of purified Tregs: CD27^+^ Tregs were then polyclonally expanded for three weeks using a modified protocol (18). Briefly, cells were stimulated for 4 days with anti-CD3/CD28 beads at a 1:1 ratio (beads:cells) in TCE medium containing 10% human AB serum, IL-2 (250 U/mL), IL-15 (10 ng/mL), TGF-β (1 ng/mL), and rapamycin (100 ng/mL). This was followed by a 3-day resting phase in TCE medium with IL-2 (50 U/mL) and 10% human AB serum. In parallel experiments, isolated CD70^+^ AS-Tregs and naïve conventional T cells (Tconv) were polyclonally expanded using repeated stimulation/resting cycles with anti-CD3/CD28 beads, IL-15, and IL-2. At each expansion cycle, cell numbers were assessed using a Countess Automated Cell Counter (Thermo Fisher Scientific) to accurately calculate the fold change in cell expansion.

At the end of the expansion protocol (day 21), cells were harvested for phenotypic characterization by flow cytometry, functional suppression and migration assays, RNA sequencing, and DNA methylation analysis. To assess phenotypic stability and resilience, day 21 expanded Tregs were restimulated for an additional week (until day 28) with anti-CD3/CD28 beads at a 1:1 ratio (beads:cells) in TCE medium containing 10% human AB serum and IL-2 (100 U/mL). To evaluate dependency on pharmacological conditioning, cells were cultured either in the continued presence of rapamycin [Rapa (+)] or in its complete absence [Rapa (–)]. In parallel, to evaluate stability under environmental stress, a cohort of the rapamycin-free cultures was additionally exposed to a pro-inflammatory cytokine cocktail containing IFN-γ, IL-1β, IL-6, and TNF-α (10 ng/mL each).

### 2.6 Flow cytometry and computational data analysis

Expanded T cells were stained for surface markers and viability using Zombie Aqua™ dye at room temperature in the dark for 20 minutes, followed by a single wash with FACS buffer. For intracellular staining, the FOXP3/Transcription Factor Staining Buffer Kit was used according to the manufacturer’s instructions. Samples were acquired on an Attune NxT Flow Cytometer (Thermo Fisher Scientific), and data were analyzed using FlowJo™ v10.10 software (BD Biosciences).

Flow cytometry data were quality-controlled using the PeacoQC plugin (23). The gating strategy sequentially isolated singlets (FSC-H vs. FSC-A), lymphocytes (SSC-A vs. FSC-A), and viable CD4^+^ T cells for subsequent marker analysis.

For high-dimensional data analysis, individual samples were concatenated within each group and subsequently merged into a single aggregate file. Dimensionality reduction and unsupervised clustering were performed using the Uniform Manifold Approximation and Projection (UMAP) v4.1.1 (24) and X-shift (25) plugins in FlowJo, respectively, using default parameters. Cluster quality was assessed using the Euclid plugin (26), which evaluates intra- and inter-cluster distances to validate population separation, using default parameters. To generate heatmaps of the clusters, the ClusterExplorer plugin in FlowJo was used.

### 2.7 Suppression assays

CD3^+^ T cells were isolated from PBMCs using the Pan T Cell Isolation Kit (Miltenyi Biotec, Germany) following the manufacturer’s instructions. Expanded Tregs (pre-labeled with CTV) were co-cultured with autologous CD3^+^ effector T cells (pre-labeled with CFSE or CTY) at various Teff: Treg ratios in TCE medium supplemented with 10% human AB serum and stimulated with irradiated allogeneic Mo-DCs at a 4:1 ratio (T cells:MoDCs). After 4 days, cells were stained with anti-CD3, anti-CD4, and anti-CD8 antibodies at room temperature in the dark for 20 minutes, washed, and acquired on an Attune NxT Flow Cytometer. Data were analyzed with FlowJo v10.10. The division index (DI) was calculated based on CFSE or CTY dilution in gated CD4^+^ or CD8^+^ effector T cells, excluding CTV-labeled Tregs. The suppression percentage was calculated using the following formula:


$$\%\;Suppression=\;\frac{\;DI\;without\;Treg-\;DI\;with\;Treg}{DI\;without\;Treg}x100$$

### 2.8 *FOXP3-*TSDR methylation analysis

DNA extraction and sodium bisulfite treatment were performed using the EZ DNA Methylation Direct Kit (Zymo Research, Irvine, USA) according to the manufacturer’s protocol. The EpiTect Whole Bisulfitome Kit (QIAGEN, Hilden, Germany) was used for amplification of the entire bisulfite-converted genome according to the manufacturer’s guidelines. Bisulfite-converted genomic DNA was subsequently amplified using the PyroMark PCR Kit (QIAGEN) following the manufacturer’s instructions. The following primers were used to amplify the Treg-specific demethylation region (TSDR) of the *FOXP3* gene:

- Set 1: p-5′-ATTTGTTTGGGGGTAGAG-3′/.
  o-5′-biotin-TATAACAACAAAACCCAAATAC-3′.
- Set 2A: p-5′-GGATGTTTTTGGGATATAG-3′/.
  o-5′-biotin-AATAAAATATCTACCCTCTTCTCT-3′.
- Set 2B: p-5′-GATTTGTTAGATTTT-3′/.
  o-5′-biotin-TTYGTTATTGAYGTTATGGYGGTYGGATGYGTYGGGTTTTATCGATATTAYGGAGGAAGAGAAGAGGGTAGATATTTTA-3′.
- Set 3: p-5′-GAGGAGAGAAGAGGGTAGATA-3′/.
  o-5′-biotin-CACCAACACCCATATCAC-3′.

All pyrosequencing reactions were carried out on a PyroMark Q24 system (QIAGEN) following the manufacturer’s guidelines.

### 2.9 Chemotaxis and migration assays

Chemotaxis assays were performed using 96-well Transwell plates (5 µm pore size). Cells (500,000 per well) were seeded in serum-free TCE medium in the upper chamber and pre-equilibrated at 37 °C and 5% CO_2_ for 10 min. Chemokine CXCL10 was added to the lower chamber at 0, 10, 100, 500, and 1000 ng/mL in serum-free TCE medium and incubated for 2 h at 37 °C. After incubation, medium from the lower wells was collected, and migrating cells were stained for phenotyping using the protocol in section 2.6. The samples were acquired on a CytoFLEX LX (Beckman Coulter, Brea, CA, USA) Cytometer. To determine the absolute number of migrating cells by flow cytometry, Precision Count Beads™ (BioLegend, San Diego, USA) were added according to the manufacturer’s instructions. The migration index was calculated using the following formula:


$$Migration\;Index=\frac{Migrated\;cells\;at\;experimental\;[chemokine]}{Migrated\;cells\;without\;chemokine\;[control]}$$

A migration index > 1 was considered indicative of chemokine-induced migration.

### 2.10 Statistical analysis of flow cytometry data

Statistical analyses were performed using GraphPad Prism software version 8.0.2 (GraphPad Software, San Diego, CA, USA). The normality of data distribution was assessed using the Shapiro–Wilk test, and homogeneity of variances for multi-group comparisons was evaluated via the Brown–Forsythe test. For two-group comparisons, statistical significance was determined using paired or unpaired Student’s t-tests for parametric data, and the Wilcoxon signed-rank or Mann–Whitney U tests for non-parametric data, as appropriate. Comparisons involving three or more groups were analyzed using ordinary one-way ANOVA, Welch’s ANOVA, or the Kruskal–Wallis test, depending on data distribution and variance assumptions. Following significant global tests, *post hoc* pairwise comparisons were performed using Tukey’s test (for ordinary ANOVA), Tamhane’s T2 test (for Welch’s ANOVA), or Dunn’s multiple comparisons test (for the Kruskal–Wallis test). Additionally, two-way ANOVA was utilized for experiments analyzing the influence of two independent variables, followed by Sidak’s or Tukey’s multiple comparisons tests, as appropriate. Data are presented as mean ± standard deviation (SD), with statistical significance defined as *p* < 0.05.

### 2.11 RNA sequencing and bioinformatics analysis

Total RNA was extracted from *ex vivo* expanded T cells using the PureLink™ RNA Mini Kit, following the manufacturer’s instructions. RNA integrity and purity were assessed by spectrophotometry and agarose gel electrophoresis prior to library preparation. High-quality RNA samples were submitted to Novogene (Beijing, China) for library construction and sequencing. Libraries were generated according to standard protocols and sequenced on the Illumina NovaSeq X Plus System, yielding 150 bp paired-end reads. Raw data underwent standard quality control using FastQC (v0.12.1) (27) and fastp (v0.23.4) (28) for adapter trimming, low-quality read filtering, and GC content assessment. Transcript-level quantification was performed using Salmon (v1.10.3) (29) using Ensembl GRCh38 human reference and gene annotation version 113. Estimated counts at the transcript level were aggregated to the gene level with tximport (v1.34.0) (30). Low-count genes were filtered prior to differential expression analysis with DESeq2 v1.50.2 (31) under a paired experimental design comparing samples collected before (untreated; n = 3) and after exposure to inflammatory cytokines (n = 3). Log_2_ fold changes were shrunk using the apeglm method to improve effect size estimation. Genes were classified as differentially expressed based on an absolute log_2_ fold change ≥ 1 and a false discovery rate (FDR) < 0.1.

## 3 Results

### 3.1 Expanded CD27^+^ AS-Tregs display a stable regulatory immunophenotype

**Figure 1:**
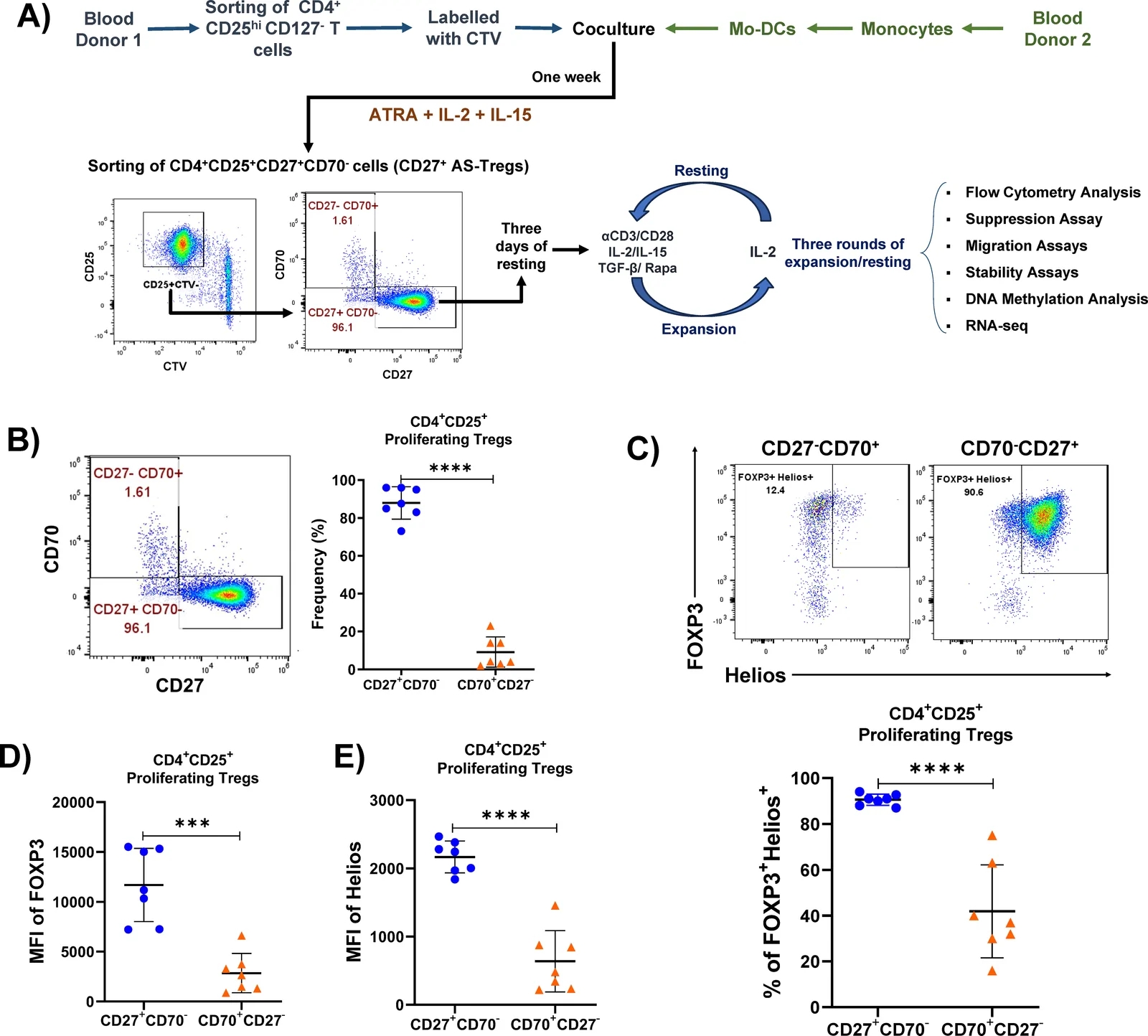
CD25^+^CD27^+^CD70^−^ alloantigen-specific Tregs exhibit high FOXP3 and Helios expression. **(A)** Schematic representation of the protocol used for the *ex vivo* expansion of AS-Tregs: CD4^+^CD25^hi^CD127^−^ T cells, labeled with CTV, were isolated by FACS and stimulated with allogeneic monocyte-derived dendritic cells (Mo-DCs) in the presence of IL-2, IL-15, and ATRA; on day 7, proliferating (CTV^low^), allospecific CD4^+^CD25^+^CD27^+^CD70^−^ T cells (CD27^+^ AS-Tregs) were purified by FACS, rested for 3 days, and subsequently subjected to four days of polyclonal expansion using anti-CD3/anti-CD28 stimulation in the presence of IL-2, IL-15, TGF-β, and rapamycin, followed by a 3-day resting phase with IL-2 alone; after three rounds of polyclonal expansion, phenotypic characterization and *in vitro* functional assays were performed. **(B–E)** Phenotypic evaluation by flow cytometry on day 7 of the Tregs: MoDCs co-culture. **(B)** Representative flow cytometry plot and cumulative data showing the frequencies of CD27^+^CD70^−^ and CD27^−^CD70^+^ subsets, highlighting that the CD27^+^CD70^−^ phenotype constitutes the major fraction of the proliferating (CTV^low^), allospecific CD25^+^ Treg population. **(C)** Representative dot plots and compiled frequencies demonstrating that the CD27^+^CD70^−^ subset maintains a significantly higher proportion of FOXP3^+^Helios^+^ cells compared to the CD27^−^CD70^+^ population. **(D, E)** Median fluorescence intensity (MFI) analysis revealing higher expression levels of both FOXP3 **(D)** and Helios **(E)** within the CD27^+^ AS-Tregs relative to their CD27^−^CD70^+^ counterparts. Data are shown as mean ± SD. Statistical analysis was performed using an unpaired t-test. ***p < 0.001, ****p < 0.0001.

To investigate the feasibility of generating an enriched population of alloantigen-specific CD25^+^CD27^+^CD70^−^ regulatory T cells (CD27^+^ AS-Tregs), we established a multistep isolation and expansion protocol, as outlined in the experimental scheme (**Figure 1A**). Initially, cells were characterized from co-cultures of isolated Tregs with allogeneic Mo-DCs. Importantly, the robust Treg proliferation observed at day 7 was dependent on specific alloantigen recognition, as the expansion cytokine cocktail (IL-2, IL-15, and ATRA) alone failed to induce proliferation in the absence of Mo-DCs (**Supplementary Figures 2A, B**). Within this proliferating (CTV^low^), alloreactive CD25^+^ Treg pool, a significantly higher proportion of CD27^+^CD70^−^ Tregs was observed compared with their CD27^−^CD70^+^ counterparts (**Figure 1B**: 87.9 ± 8.5% vs. 9.2 ± 7.9%; *p* < 0.001). Notably, these allospecific CD27^+^CD70^−^ Tregs contained a significantly higher proportion of FOXP3^+^Helios^+^ cells than the CD27^−^CD70^+^ subset (**Figure 1C**: 90.5 ± 2.5% vs. 41.9 ± 20.3%; *p* < 0.0001). Consistent with this, the mean fluorescence intensity (MFI) of both FOXP3 and Helios was significantly elevated in the CD27^+^CD70^−^ population compared to their CD27^−^CD70^+^ counterparts (**Figures 1D, E**).

Following the 7-day co-culture with allogeneic Mo-DCs, CD27^+−^ AS-Tregs were isolated by flow cytometry. Immediately post-sorting, these cells exhibited high purity and a stable regulatory phenotype, characterized by high frequencies of CD25^+^FOXP3^+^ (97.0 ± 1.5%), Helios^+^ (85.9 ± 2.1%), CTLA-4^+^ (99.6 ± 0.3%), and CD39^+^ (72.7 ± 18.2%) cells. Conversely, frequencies of the markers TIM-3 (4.2 ± 2.3%) and CD73 (1.9 ± 1.5%) were minimal (**Supplementary Figure 2C**).

**Figure 2:**
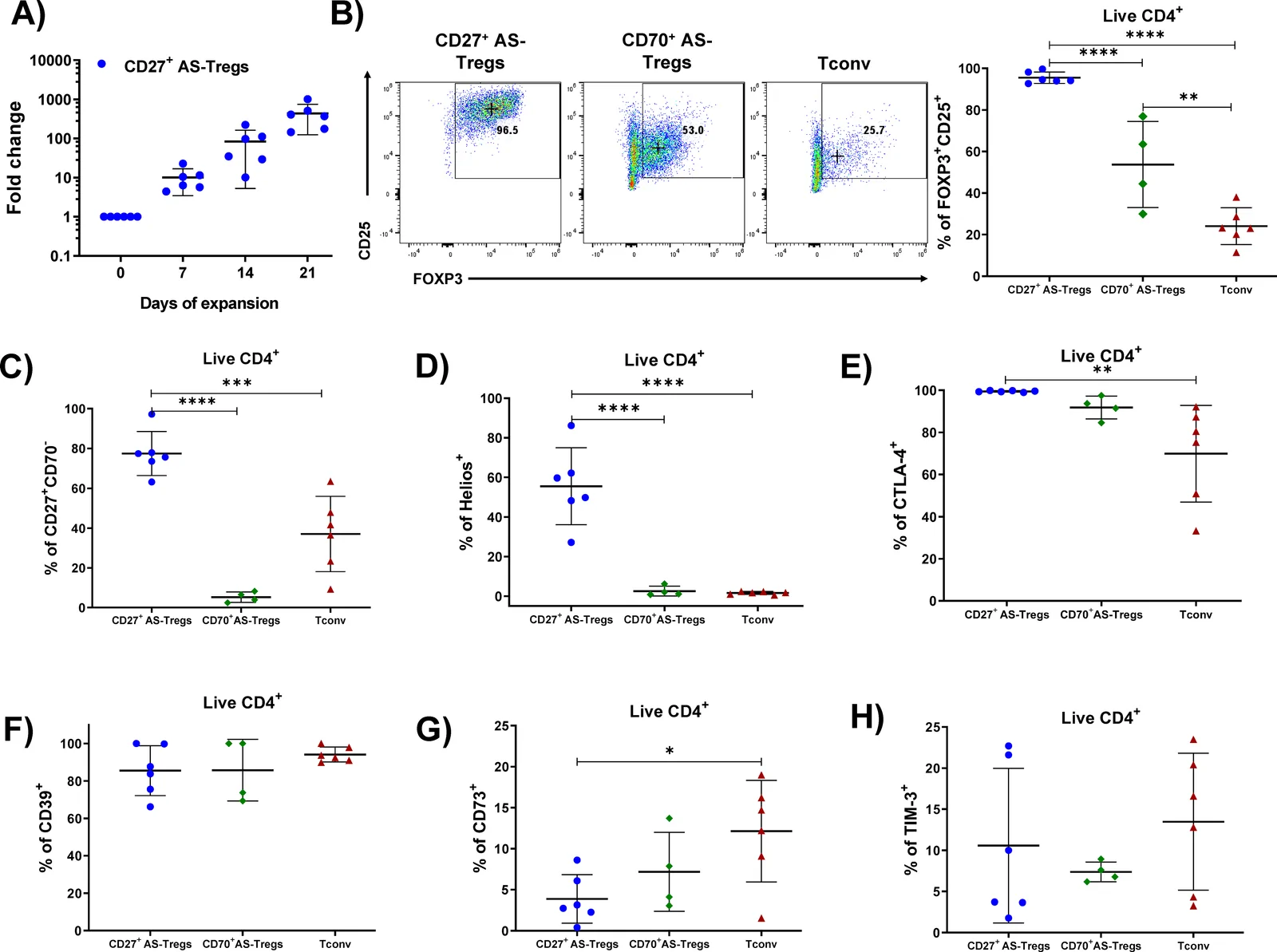
CD27^+^ allospecific Tregs expand and retain a regulatory phenotype after prolonged *ex vivo* culture. Allospecific Tregs, sorted as CD25^+^CD27^+^CD70^−^ (CD27^+^ AS-Tregs) and CD25^+^CD27^−^CD70^+^ (CD70^+^ AS-Tregs), alongside conventional CD4^+^CD25^−^CD45RA^+^ T cells (Tconv), were isolated and polyclonally expanded for three weeks. **(A)** Fold expansion curve demonstrates that CD27^+^ AS-Tregs achieve a substantial proliferative yield, expanding on average 434-fold by day 21. **(B–H)** Phenotypic assessment by flow cytometry after the 3-week expansion. **(B)** Representative flow cytometry plots and cumulative data highlighting that expanded CD27^+^ AS-Tregs maintain high co-expression of CD25 and FOXP3 (>95%), whereas CD70^+^ AS-Tregs and Tconv subsets exhibit significant loss of FOXP3. **(C, D)** Compiled frequencies illustrating that CD27^+^ AS-Tregs selectively preserve the CD27^+^CD70^−^ signature **(C)** and the lineage-stability marker Helios **(D)**, both of which are significantly reduced in the comparator groups. **(E)** CD27^+^ AS-Tregs uniformly retain expression of the essential co-inhibitory molecule CTLA-4 (~100%), which is significantly higher than in Tconv cells. **(F–H)** Assessment of additional functional markers reveals comparable, high expression of the ectonucleotidase CD39 across all subsets **(F)**, along with the distinct frequencies of CD73^+^ **(G)** and TIM-3^+^ **(H)** cells. Data are shown as mean ± SD. Statistical analysis was performed using a one-way ANOVA test. *p < 0.05, **p < 0.01, ***p < 0.001, ****p < 0.0001.

Subsequently, the sorted CD27^+^ AS-Tregs, CD70^+^ AS-Tregs, and Tconv cells underwent a 3-week expansion phase. By day 21, CD27^+^ AS-Tregs demonstrated a robust mean expansion of 434-fold relative to their initial sorted cell count (**Figure 2A**). Following this prolonged expansion, the functional phenotype was thoroughly evaluated. The proportion of CD25^+^FOXP3^+^ cells remained significantly higher in the CD27^+^ AS-Treg cultures compared to both CD70^+^ AS-Tregs and Tconv controls (**Figure 2B**: 95.5 ± 2.8% vs. 53.7 ± 20.8% and 24.1 ± 8.9%, respectively). Furthermore, the CD27^+^ AS-Tregs showed a significantly higher frequency of the targeted CD27^+^CD70^−^ phenotype (**Figure 2C**: 77.5 ± 11.1% vs. 5.3 ± 2.6% in CD70^+^ AS-Tregs and 37.0 ± 19.0% in Tconv). Consistent with a regulatory profile, these findings correlated with a significantly increased proportion of Helios^+^ cells within the CD27^+^ AS-Treg population compared to the control groups (**Figure 2D**: 55.6 ± 19.4% vs. 2.6 ± 2.5% and 42.6 ± 19.2%, respectively).

To evaluate the phenotypic dynamics of the expanded AS-Treg at a per-cell level, we longitudinally tracked the MFI of FOXP3, CD27, and CD70 over a 21-day *ex vivo* expansion period (**Supplementary Figure 3**). The MFI profiles of the Treg master transcription factor FOXP3 (**Supplementary Figure 3A**), the co-stimulatory receptor CD27 (**Supplementary Figure 3B**), and its ligand CD70 (**Supplementary Figure 3C**) on CD27^+^ AS-Tregs showed stable trends, maintaining consistent intensity levels from Day 0 through Day 21. Statistical analysis confirmed that there were no significant changes in the per-cell density of any of these three proteins across the investigated time points (*p* > 0.05).

Furthermore, expanded CD27^+^ AS-Tregs maintained a high frequency of CTLA-4^+^ cells, significantly greater than expanded Tconv, though comparable to CD70^+^ AS-Tregs (**Figure 2E**: 99.5 ± 0.3% vs. 69.9 ± 23.0% and 91.8 ± 5.4%, respectively). All three cell populations exhibited similarly high proportions (>85%) of CD39^+^ cells (**Figure 2F**: 85.5 ± 13.4%, 85.8 ± 2.5%, and 94.6 ± 4.0%, respectively). Conversely, surface expression of CD73 (**Figure 2G**: range 3.9–12.1%) and TIM-3 (**Figure 2H**: range 7.4–13.5%) remained uniformly low (<25%) across all groups.

**Figure 3:**
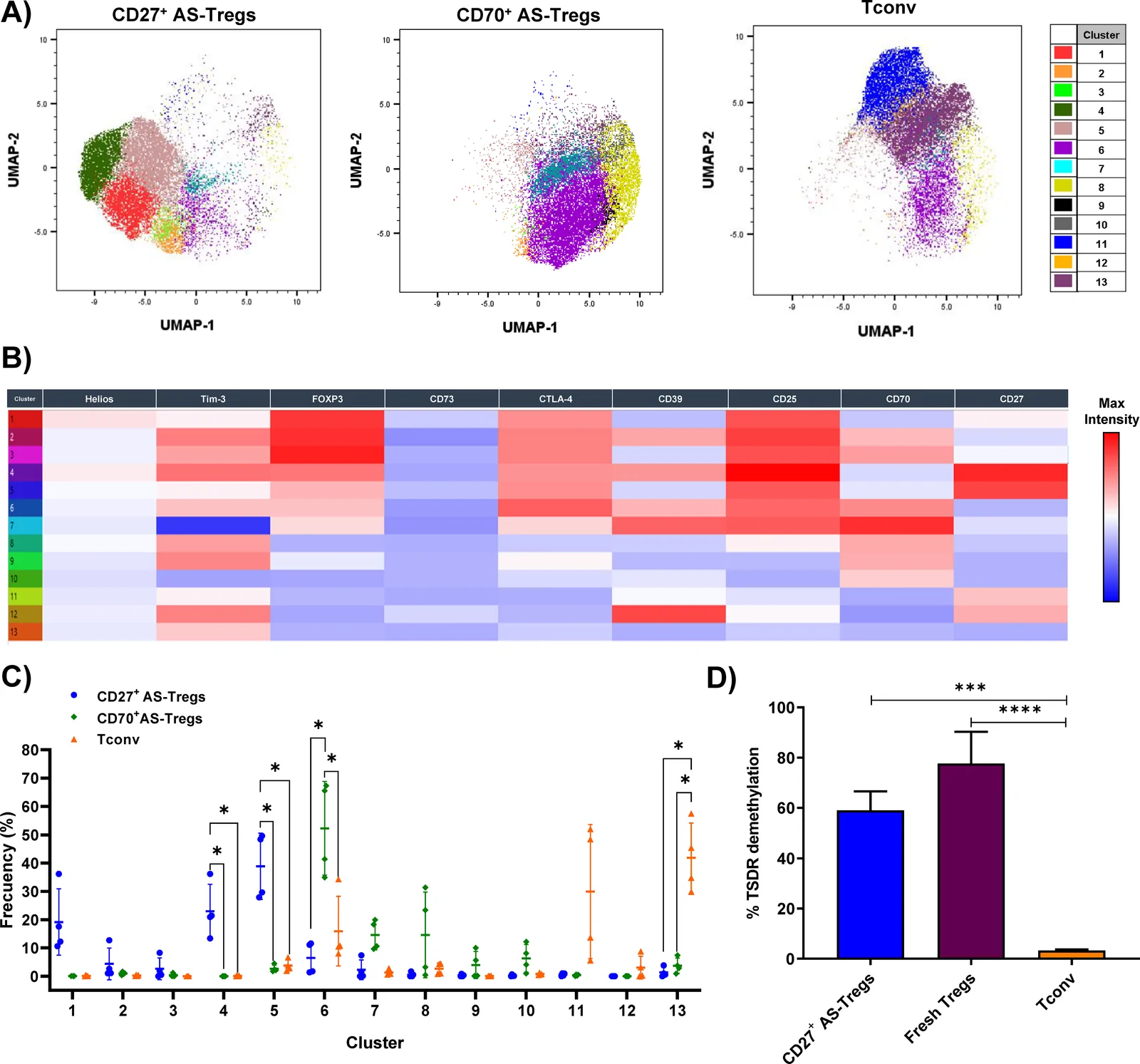
Expanded CD27^+^ AS-Tregs maintain a regulatory multidimensional signature and *FOXP3*-TSDR demethylation. **(A–C)** Unsupervised dimensionality reduction and clustering analysis of flow cytometry data acquired on day 21 of expansion (n=4 per group). **(A)** UMAP projections, generated from a concatenated file integrating all experimental samples, reveal profound phenotypic segregation among the expanded subsets, with CD27^+^ AS-Tregs clustering distinctly apart from CD70^+^ AS-Tregs and Tconv cells. **(B)** Heatmap detailing the marker expression intensity across the 13 identified clusters. **(C)** Frequencies of each cluster within the distinct T cell subsets. Individual data points represent independent biological replicates, reflecting the sample-level statistical analysis and confirming the selective and significant enrichment of clusters in the CD27^+^ AS-Tregs (clusters 4 and 5), CD70^+^ AS-Tregs (cluster 6) and Tconv (cluster 13) (n=4 per group). **(D)** Targeted CpG methylation analysis of the *FOXP3* Treg-specific demethylated region (TSDR) (n=3 per group). Expanded CD27^+^ AS-Tregs retain substantial demethylation levels that closely mirror those of freshly isolated CD4^+^CD25^hi^CD127^−^ Tregs, in stark contrast to the fully methylated profile of Tconv cells. Data are shown as mean ± SD. Statistical analyses were performed using a two-way ANOVA **(C)** and a one-way ANOVA **(D)**. *p < 0.05, ***p < 0.001, ****p < 0.0001.

To further resolve the phenotypic landscape of the expanded cells, we performed high-dimensional flow cytometry analysis. UMAP dimensionality reduction revealed that expanded CD27^+^ AS-Tregs, CD70^+^ AS-Tregs, and Tconv cells occupy distinct topological spaces, underscoring their unique protein expression signatures (**Figures 3A, B**; **Supplementary Figure 4A**). Specifically, CD27^+^ AS-Tregs were significantly enriched in clusters 4 and 5 (**Figure 3C**). These representative clusters define a robust suppressive profile: cluster 4 (comprising 23.0 ± 9.6% of CD27^+^ AS-Tregs, but <0.2% of the other subsets) is characterized by the co-expression of Helios, FOXP3, CTLA-4, and CD39 (**Figure 3B**; **Supplementary Figure 4B**). Meanwhile, cluster 5 (38.9 ± 11.7% of CD27^+^ AS-Tregs, with minimal contribution from other groups) exhibits a similar core regulatory profile but lacks CD39 and TIM-3 expression. In contrast, cluster 6 was primarily enriched for CD70^+^ AS-Tregs (52.3 ± 16.5% vs. <16% in other subsets) and displayed a distinct phenotype characterized by low expression of FOXP3, Helios, and CD27, despite retaining CTLA-4 and CD39 (**Figures 3B, C**; **Supplementary Figure 4B**). Finally, cluster 13, which was significantly enriched for Tconv cells (41.9 ± 12.3%), lacked all core Treg markers (Helios, FOXP3, and CTLA-4) (**Figures 3B, C**; **Supplementary Figure 4B**).

### 3.2 CD27^+^ AS-Tregs exhibit epigenetic stability and a conserved transcriptional signature

To confirm that this phenotypic stability was underpinned by epigenetic programming, we analyzed the Treg-specific demethylated region (TSDR) of the *FOXP3* locus, also known as conserved non-coding sequence 2 (CNS2). In agreement with the protein expression data, CD27^+^ AS-Tregs expanded for three weeks exhibited a TSDR demethylation level of 59.0 ± 7.6% (**Figure 3D**). In comparison, freshly isolated Tregs showed a demethylation level of 77.6 ± 12.7%, while expanded Tconv cells displayed 3.3 ± 0.4% (**Figure 3D**).

**Figure 4:**
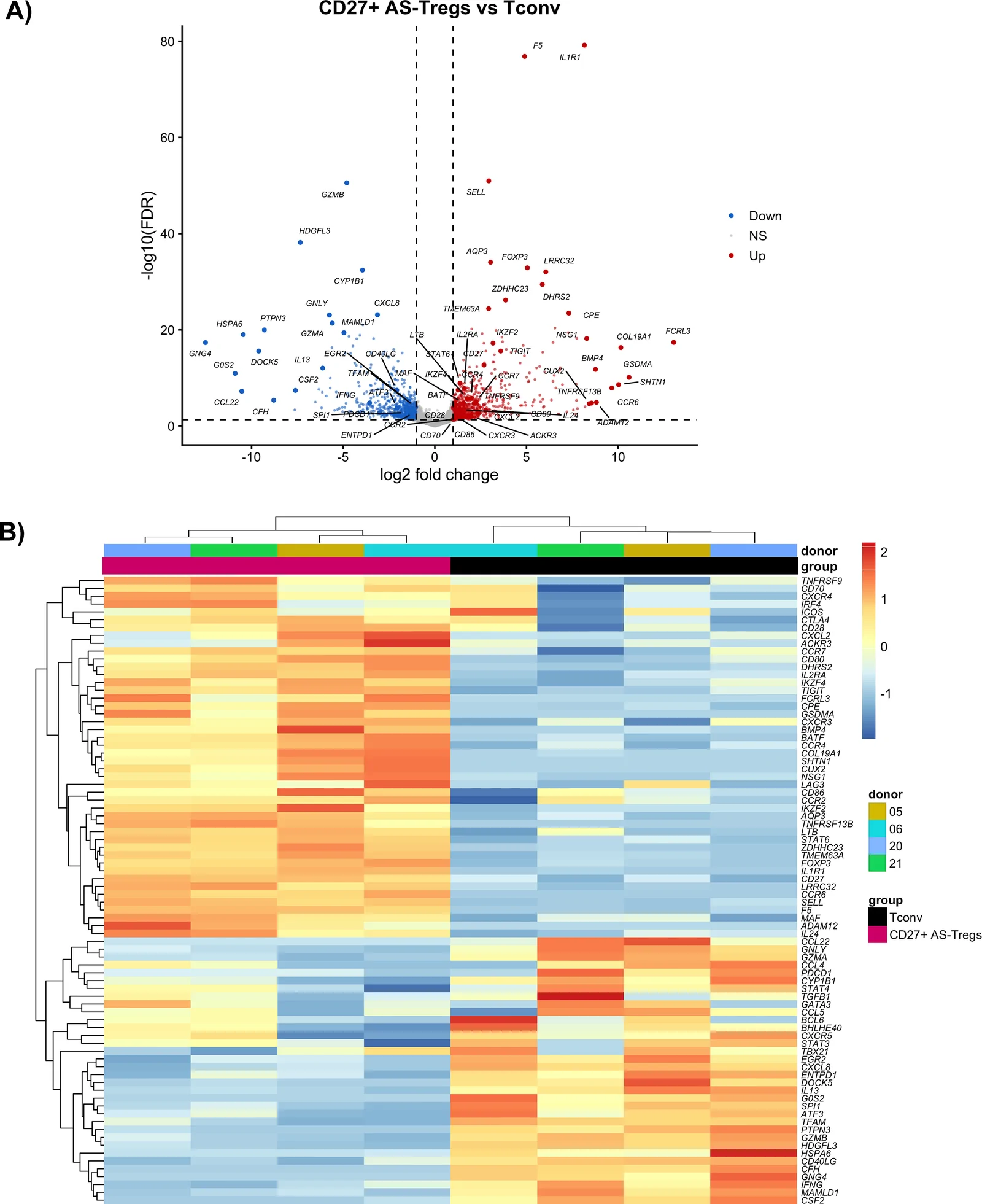
Long-term expanded CD27^+^ AS-Tregs exhibit a regulatory transcriptome. **(A, B)** Global transcriptional profiling by bulk RNA sequencing performed on day 21 of expansion. **(A)** Volcano plot illustrating robust differential gene expression between expanded CD27^+^ AS-Tregs (n=4) and Tconv cells (n=4), highlighting the significant upregulation of core Treg lineage markers (including *FOXP3*, *IL2RA* (CD25), *CD27, CTLA4*, *TIGIT*, *LRRC32* (GARP), *TNFRSF9* (CD137), *IKZF2* (Helios), *IKZF4* (Eos) and *BATF*) and the active repression of inflammatory transcripts (*IFNG*, *IL13*, *CXCL8*, *CCL22*, *CSF2* (GM-CSF), *GZMA* and *GZMB*) in CD27^+^ AS-Tregs. **(B)** Hierarchical clustering and heatmap of the top differentially expressed genes, showing a distinct, prototypical regulatory signature in CD27^+^ AS-Tregs that segregates from the Tconv profile. The complete list of differentially expressed genes is provided in **Supplementary Tables 1–3**.

Transcriptomic profiling revealed that CD27^+^ AS-Tregs expanded for 21 days exhibit a distinct and coherent gene expression program, consistent with a regulatory identity and suppressive function when compared to Tconv cells. Volcano plot analysis (**Figure 4A**; **Supplementary Tables 1–2**) identified a broad set of differentially expressed genes (DEGs). Specifically, CD27^+^ AS-Tregs showed preferential upregulation of Treg-associated and immunoregulatory genes, including *FOXP3*, *IL2RA* (CD25), *CD27, CTLA4*, *TIGIT*, *LRRC32* (GARP), and *TNFRSF9* (CD137), alongside key transcriptional regulators such as *IKZF2* (Helios), *IKZF4* (Eos) and *BATF*. In stark contrast, expanded Tconv cells displayed a transcriptomic profile enriched for genes associated with inflammatory responses, effector T cell differentiation, and cytokine signaling, notably *IFNG*, *IL13*, *CXCL8*, *CCL22*, *CSF2* (GM-CSF), *GZMA* and *GZMB*.

To further resolve these distinct transcriptional landscapes, heatmap analysis of selected immunologically relevant DEGs (**Figure 4B**) demonstrated robust hierarchical segregation between the CD27^+^ AS-Treg and Tconv populations, clustering independently of underlying donor-to-donor variability. The CD27^+^ AS-Treg replicates clustered tightly together, defined by the coordinated expression of genes directly linked to suppressive function and Treg lineage stability. Conversely, Tconv samples were predominantly characterized by the heightened expression of inflammatory mediators, chemokines, and effector-associated genes (**Supplementary Table 3**).

### 3.3 Expanded CD27^+^ AS-Tregs exert antigen-specific suppression of effector T cells

**Figure 5:**
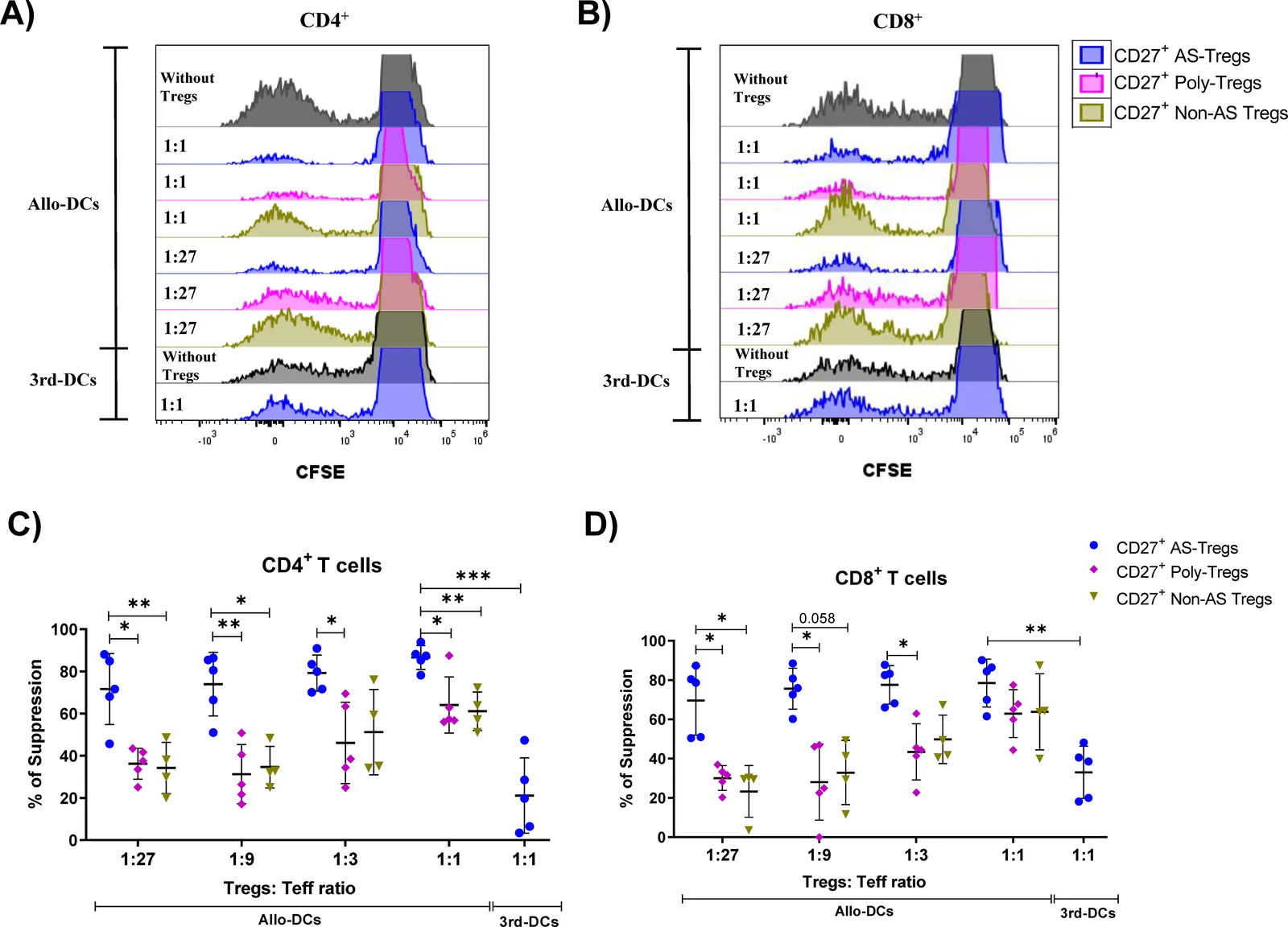
Expanded CD27^+^ AS-Tregs exert alloantigen-specific immunosuppression. **(A, B, A–D)** *In vitro* suppression assays conducted on day 21 of expansion. **(A, B)** Representative flow cytometry histograms demonstrating the profound inhibition of autologous effector CD4^+^ **(C)** and CD8^+^ **(D)** T cell proliferation (assessed by CFSE dilution) by CD27^+^ AS-Tregs (blue histograms) compared to both polyclonal CD27^+^ Tregs (purple histograms) and the internal non-proliferating control, CD27^+^ Non-AS Tregs (olive histograms). Baseline proliferation of effector T cells stimulated with specific Allo-DCs or third-party MoDCs (3rd-DCs) in the absence of Tregs is shown for reference (gray histograms), along with the minimal suppression exerted by AS-Tregs against 3rd-DCs at a 1:1 ratio (bottom blue histograms). **(C, D)** Quantitative analysis of suppressive capacity against CD4^+^ **(C)** and CD8^+^ **(D)** effector T cells across multiple Treg: Teff ratios. Expanded CD27^+^ AS-Tregs (blue circles) display a higher, dose-dependent suppressive advantage over both polyclonal CD27^+^ Tregs (purple diamonds) and CD27^+^ Non-AS Tregs (olive inverted triangles) against specific allogeneic MoDCs (Allo-DCs). This robust suppression is stringently alloantigen-specific, as evidenced by the significantly reduced suppressive activity when CD27^+^ AS-Tregs are challenged with 3rd-DCs at a 1:1 ratio. Data are shown as mean ± SD. Statistical significance for Treg potency against specific Allo-DCs among the three groups was determined using a Two-way ANOVA. Suppression by CD27^+^ AS-Tregs at a 1:1 ratio against Allo-DCs versus 3rd-DCs was compared using a paired Student’s t-test. *p < 0.05, **p < 0.01, ***p < 0.001.

Subsequently, we assessed whether the preserved immunophenotype of expanded CD27^+^ AS-Tregs translated into an enhanced capacity to suppress effector T cell proliferation *in vitro*. To rigorously evaluate this, we compared their suppressive potency against two distinct control populations expanded under the exact same culture conditions: polyclonal CD27^+^ Tregs and a stringent internal control, termed CD27^+^ Non-AS Tregs (defined as the non-proliferating CTV^+^ fraction isolated from the identical Treg-MoDC co-culture). Suppression assays confirmed that CD27^+^ AS-Tregs efficiently inhibited the proliferation of both CD4^+^ and CD8^+^ effector T cells (**Figures 5A–D**). Importantly, while the suppressive capacity of standard polyclonal and Non-AS Tregs declined rapidly at lower concentrations, CD27^+^ AS-Tregs exhibited a sustained suppressive advantage across a wide range of Treg: Teff ratios. Notably, even when titrated down to a 1:27 ratio, CD27^+^ AS-Tregs preserved a robust suppressive capacity, whereas both control populations lost nearly all ability to inhibit proliferation.

Furthermore, to confirm that this robust anti-proliferative activity was strictly donor-specific, cells were challenged with third-party dendritic cells (3rd-DCs). Crucially, CD27^+^ AS-Tregs failed to exert cross-suppression against third-party targets, demonstrating a significant reduction in their ability to suppress T cell proliferation compared to their robust response against the specific Allo-DCs (**Figures 5C, D**).

To ensure that the enhanced suppressive capacity of AS-Tregs was inherently driven by specific TCR engagement rather than a loss of regulatory identity in the comparator groups, we immunophenotyped both control populations utilized in the functional assays (**Supplementary Figure 5**). Notably, both the expanded polyclonal Tregs and the internal Non-AS Treg controls maintained stable and statistically indistinguishable regulatory profiles. Both populations expressed equivalently high levels of key lineage and suppressive markers—including CD25^+^FOXP3^+^, CD27^+^CD70^−^, Helios^+^, CTLA-4^+^, and CD39^+^.

### 3.4 Expanded CD27^+^ AS-Tregs express chemokine receptors associated with tissue and lymphoid homing

**Figure 6:**
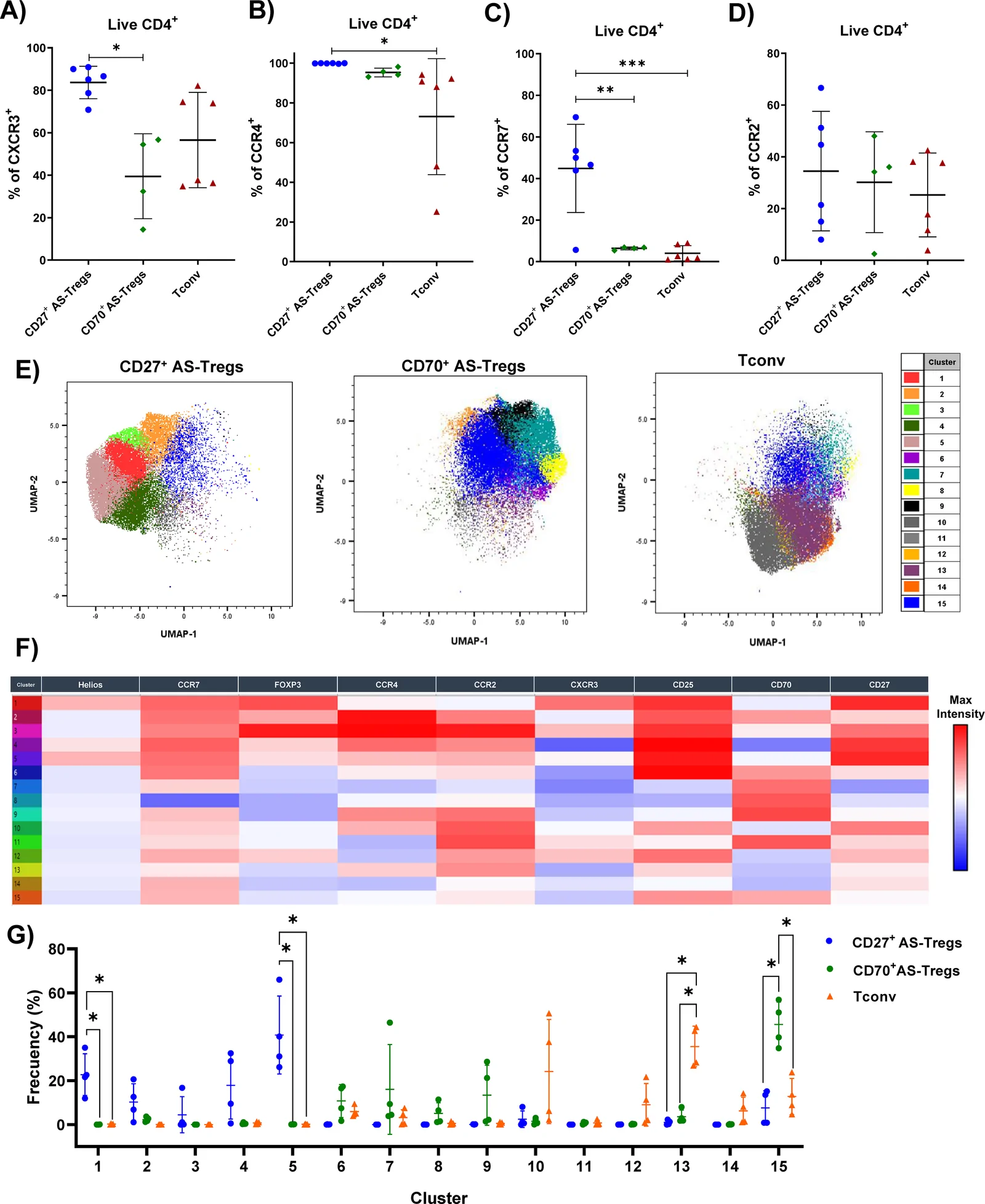
Expanded CD27^+^ AS-Tregs maintain a dual-homing chemokine receptor profile associated with lymphoid tissue and allograft migration. **(A–D)** Flow cytometry analysis performed on day 21 of *in vitro* expansion. Expanded CD27^+^ AS-Tregs display significantly elevated frequencies of the tissue- and allograft-homing receptors CXCR3 **(A)** and CCR4 **(B)**, as well as the lymphoid-homing receptor CCR7 **(C)**, compared to CD70^+^ AS-Tregs and Tconv cells. The frequency of CCR2^+^ cells **(D)** remained comparable across all evaluated subsets. **(E)** Unsupervised UMAP dimensionality reduction reveals segregated, subset-specific chemokine receptor landscapes among the expanded CD27^+^ AS-Tregs, CD70^+^ AS-Tregs, and Tconv cells. **(F)** Heatmap detailing marker expression intensity across the 15 identified clusters. **(G)** Frequencies of each cluster within the distinct T cell subsets. Individual data points represent independent biological replicates, reflecting the sample-level statistical analysis and confirming the selective and significant enrichment of specific clusters in the CD27^+^ AS-Tregs (clusters 1 and 5), CD70^+^ AS-Tregs (cluster 15), and Tconv cells (cluster 13) (n=4 per group). Data are shown as mean ± SD. Statistical analysis was performed using a one-way ANOVA or Kruskal-Wallis test **(A–D)** as appropriate, and a two-way ANOVA **(G)**. *p < 0.05, **p < 0.01, ***p < 0.001.

To evaluate the migratory potential of the expanded cells, we analyzed the surface expression of key chemokine receptors at day 21 of expansion. CD27^+^ AS-Tregs displayed a significantly higher percentage of CXCR3^+^ cells compared to the CD70^+^ AS-Treg subset (**Figure 6A**: 83.7 ± 7.7% vs. 39.5 ± 20.0%, respectively). Furthermore, CD27^+^ AS-Tregs were uniformly positive for CCR4, showing significant enrichment compared to Tconv cells (**Figure 6B**: 99.9 ± 0.1% vs. 73.1 ± 29.2%). Notably, CD27^+^ AS-Tregs retained a significantly higher proportion of CCR7^+^ cells compared to both comparison groups (**Figure 6C**: 44.8 ± 21.2% vs. <7.0% in both CD70^+^ AS-Tregs and Tconv). In contrast, all three cell populations exhibited intermediate and comparable levels of CCR2, with no significant differences observed among the groups (**Figure 6D**: range 25.3–34.5%).

UMAP analysis further highlighted the distinct migratory profiles of these populations (**Figure 6E–G**; **Supplementary S6A**). CD27^+^ AS-Tregs were significantly enriched in clusters 1 (22.7 ± 9.5%) and 5 (40.8 ± 17.8%), with a negligible contribution from CD70^+^ AS-Tregs or Tconv cells (< 0.3% in both clusters) (**Figure 6G**). Phenotypically, these CD27^+^-dominated clusters exhibited a “dual-homing” signature, characterized by the co-expression of lymphoid (CCR7 and/or CCR2) and inflammatory (CXCR3 and/or CCR4) trafficking receptors, alongside robust expression of FOXP3, Helios, and CD27 (**Figure 6F**; **Supplementary Figure 6B**). In contrast, Tconv cells and CD70^+^ AS-Tregs predominantly localized to clusters 13 (35.6 ± 9.3% of Tconv) and 15 (45.6 ± 10.1% of CD70^+^ AS-Tregs), respectively (**Figure 6G**). Both of these clusters displayed a distinct non-Treg phenotype (Helios^−^FOXP3^−^) with low expression of CCR7 and CXCR3. Furthermore, cluster 13 was distinguished by a CCR4^low^CCR2^+^ profile, whereas cluster 15 was defined by high CD70 expression paired with low levels of both CCR4 and CCR2 (**Figure 6F** and **Supplementary Figure 6B**).

### 3.5 CD27^+^ AS-Tregs exhibit CXCR3-dependent migration

**Figure 7:**
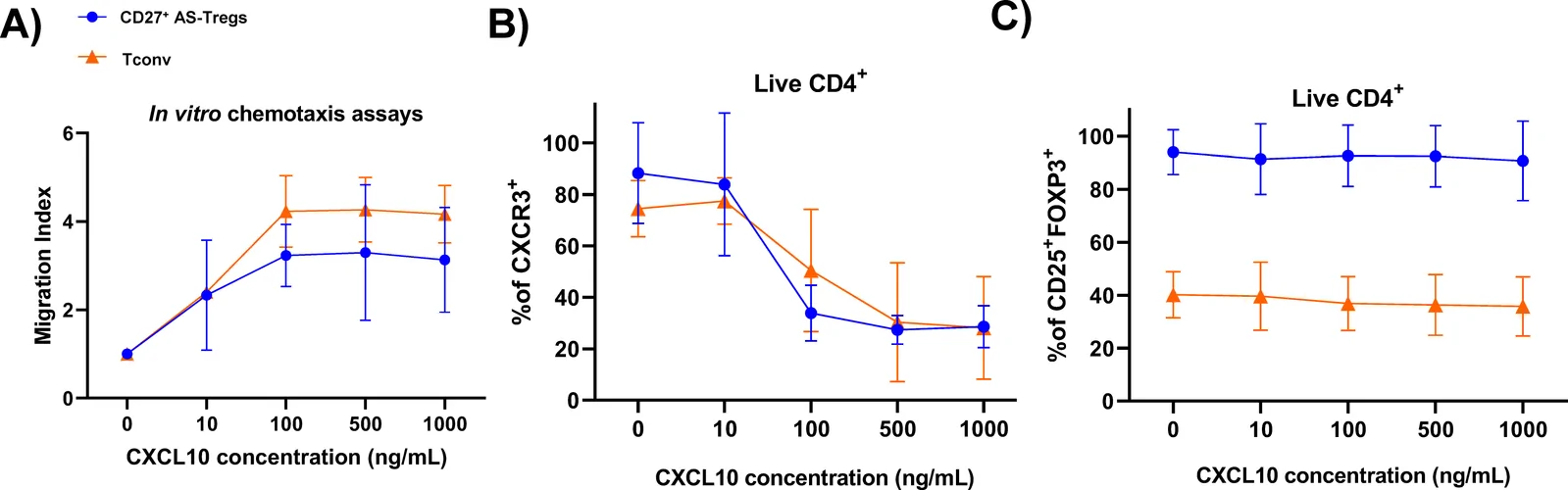
CD27^+^ AS-Tregs exhibit chemotaxis toward CXCL10 while maintaining their regulatory identity. **(A)** *In vitro* chemotaxis assays performed on day 21 of expansion demonstrate that both CD27^+^ AS-Tregs (n=3) and activated Tconv cells (n=3) possess functional migratory capacity, displaying a robust, dose-dependent migration index in response to increasing concentrations of CXCL10. **(B, C)** Phenotypic evaluation of the actively migrating populations by flow cytometry. **(B)** Both CD27^+^ AS-Tregs and Tconv subsets exhibit a dose-dependent reduction in the frequency of surface CXCR3^+^ cells, a dynamic consistent with expected ligand-induced receptor internalization following active chemotaxis. **(C)** Migrating CD27^+^ AS-Tregs maintained a uniformly high and significantly superior proportion of CD25^+^FOXP3^+^ cells across all CXCL10 concentrations compared to Tconv cells. Data are shown as mean ± SD. Statistical analysis was performed using a two-way ANOVA. *p < 0.05.

To determine whether the expression of these homing receptors translates into functional activity, we evaluated the chemotactic potential of expanded CD27^+^ AS-Tregs. To this end, *in vitro* chemotaxis assays were performed using CXCL10, the primary ligand for CXCR3. Expanded CD27^+^ AS-Tregs exhibited a concentration-dependent chemotactic response, initiating active migration at doses as low as 10 ng/mL (Migration Index = 2.33 ± 2.1) and reaching a maximal plateau at concentrations ≥100 ng/mL (Migration Index range: 3.23–3.30) (**Figure 7A**). Notably, the migratory capacity of CD27^+^ AS-Tregs was efficient and followed a dynamic trajectory similar to that of activated Tconv cells, with the latter reaching a maximal plateau at concentrations ≥100 ng/mL (Migration Index range: 4.17–4.27) (**Figure 7A**).

To further corroborate the specificity of this chemokine-receptor axis, we analyzed the surface expression of CXCR3 on the transmigrated cell fractions. Both CD27^+^ AS-Tregs and Tconv cells demonstrated a pronounced, dose-dependent reduction in surface CXCR3 positivity—dropping sharply from baseline levels of ~80–90% to approximately 30% at CXCL10 doses ≥100 ng/mL (**Figure 7B**). This progressive downregulation is a classic hallmark of active, ligand-induced receptor internalization.

Finally, we evaluated the phenotype of these cells following active migration to ensure that the chemotactic process did not alter their lineage identity. Remarkably, the transmigrated fraction of CD27^+^ AS-Tregs strictly preserved its canonical regulatory phenotype. The proportion of CD25^+^FOXP3^+^ cells remained consistently stable at >90% across all evaluated CXCL10 concentrations, in sharp contrast to Tconv cells, which maintained their expected lower baseline frequencies (<40%) (**Figure 7C**).

### 3.6 Phenotypic stability of expanded CD27^+^ AS-Tregs is maintained following rapamycin withdrawal

To determine whether the robust stability observed in expanded CD27^+^ AS-Tregs strictly required continuous pharmacological support, expanded cells were subjected to re-stimulation and cultured for an additional week in either the continued presence (Rapa (+)) or the complete absence (Rapa (–)) of rapamycin (**Supplementary Figure 7**). Detailed immunophenotyping revealed that rapamycin withdrawal did not compromise core lineage integrity. Lineage-defining protein expression levels (MFI) remained preserved without the drug, including stable expression of FOXP3 (**Supplementary Figure 7A**), CD25 (**Supplementary Figure 7B**), and CD27 (**Supplementary Figure 7C**) alongside low CD70 levels (**Supplementary Figures 7C, D**), and unaltered expression of the lineage-stability marker Helios (**Supplementary Figure 7E**).

Interestingly, while the expression of co-inhibitory molecules such as CTLA-4 (**Supplementary Figure 7F**) and TIM-3 (**Supplementary Figure 7G**) remained stable regardless of the culture conditions, the continued presence of rapamycin (Rapa (+)) drove a significant upregulation in the per-cell protein expression (MFI) of the functionally critical ectonucleotidases CD39 (**Supplementary Figure 7H**) and CD73 (**Supplementary Figure 7I**).

### 3.7 CD27^+^ AS-Tregs preserve phenotypic and functional stability under pro-inflammatory conditions

**Figure 8:**
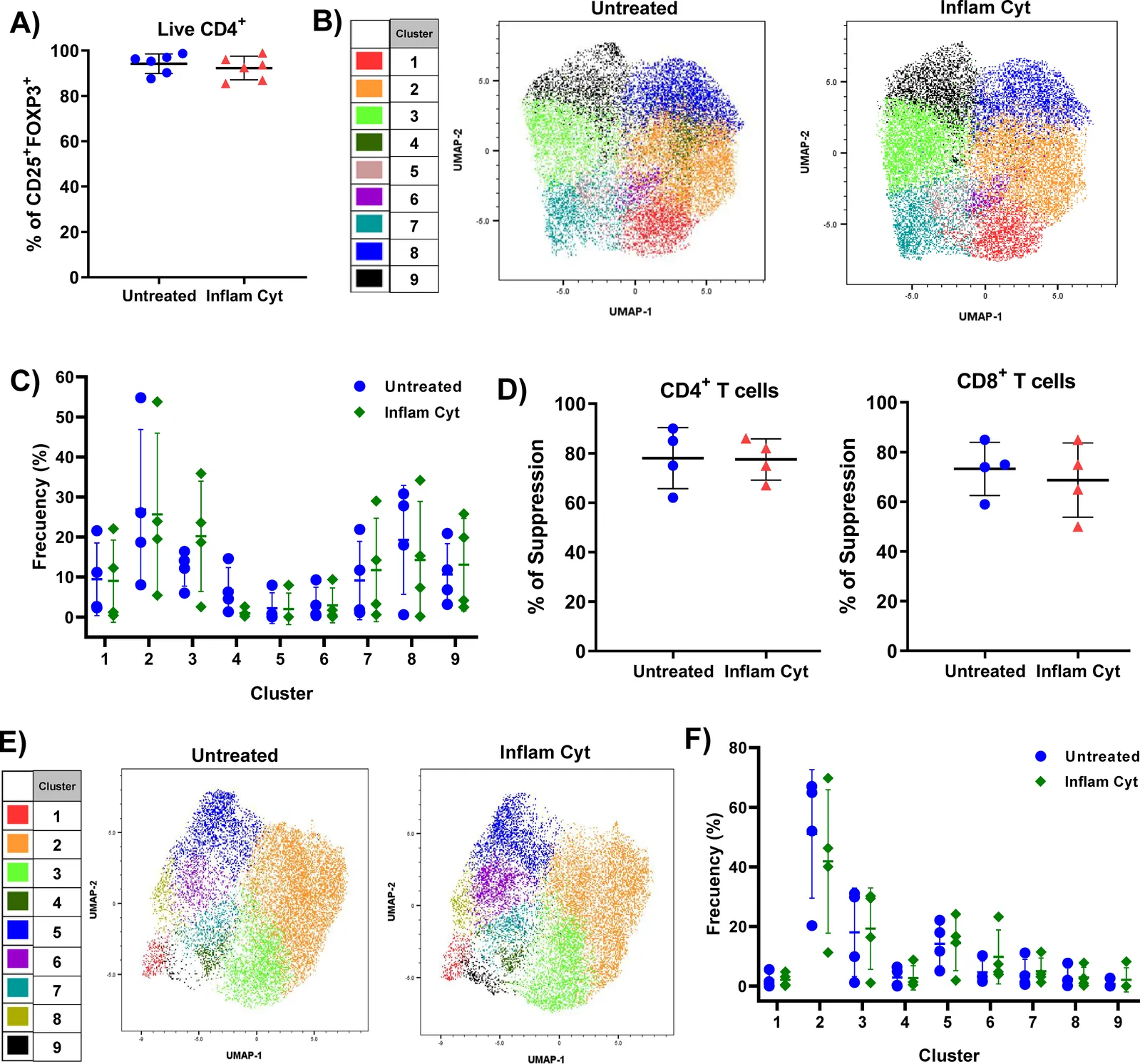
CD27^+^ AS-Tregs preserve their phenotypic, functional, and migratory identity under inflammatory conditions. Following 21 days of expansion, CD27^+^ AS-Tregs were cultured for an additional week either in the presence of an inflammatory cytokine cocktail (IFN-γ, IL-1β, IL-6, and TNF-α) or in standard control medium (Untreated). **(A)** Flow cytometry quantification demonstrates that the CD25^+^FOXP3^+^ regulatory identity remains stable, showing no significant decline following exposure to the inflammatory milieu. **(B, C)** Unsupervised high-dimensional analysis of immunoregulatory markers. UMAP projections **(B)** and frequencies of each cluster within each condition **(C)** show that the regulatory landscape of CD27^+^ AS-Tregs is resistant to inflammatory stimulation, displaying comparable profiles between cytokine-stimulated and control conditions. **(D)** *In vitro* suppression assays confirm that the capacity of CD27^+^ AS-Tregs to inhibit both CD4^+^ and CD8^+^ effector T cell proliferation is maintained after inflammatory challenge. **(E, F)** High-dimensional evaluation of the chemokine receptor repertoire. UMAP projections **(E)** and frequencies of each cluster within each condition **(F)** confirm that the dual-homing migratory signature of CD27^+^ AS-Tregs remains consistent under inflammatory conditions. For both **(C, F)**, individual data points represent independent biological replicates (n=4 per group), reflecting the sample-level statistical analysis. Data are shown as mean ± SD. Statistical analysis was performed using a paired Student’s t-test **(A, D)** or a two-way ANOVA **(C, F)**. No statistically significant differences were observed across any of the assessed parameters.

Given that adoptive cell therapy requires Tregs to function effectively within inflammatory environments, we evaluated the phenotype of our 3-week expanded CD27^+^ AS-Tregs by challenging them with a pro-inflammatory cytokine cocktail (IFN-γ, IL-6, IL-1β, and TNF-α) for one additional week. Flow cytometric analysis revealed consistent stability in the presence of this inflammatory microenvironment. Both the proportion of CD25^+^FOXP3^+^ cells (**Figure 8A**: 94.2 ± 4.3% vs. 92.3 ± 5.2% in controls) and overall FOXP3 expression levels (MFI) (**Supplementary Figure 8A**: 13,764 ± 9,744 vs. 12,820 ± 11,114) remained robust and unchanged.

Furthermore, although a slight reduction in the frequency of the CD27^+^CD70^−^ subset was observed following the inflammatory challenge (61.1 ± 13.6% vs. 71.0 ± 16.0% in controls), the expression of core regulatory and functional molecules—such as Helios (38.3 ± 13.6% vs. 40.3 ± 11.6%), CD39 (60.1 ± 41.0% vs. 55.6 ± 39.4%), and CTLA-4 (>99.5% in both conditions)—was preserved at high levels, entirely comparable to standard control conditions (**Supplementary Figures 8B–E**). Finally, the proportions of cells expressing CD73 and TIM-3 remained uniformly low (<20%), with no significant upregulation induced by the inflammatory cytokines (**Supplementary Figures 8F, G**).

Consistent with these findings, high-dimensional flow cytometry analysis demonstrated that exposure to inflammatory cytokines did not alter the overall topological distribution of the cells (**Supplementary Figure 8H**) or the phenotypic composition of the core immunoregulatory clusters (**Figures 8B, C**; **Supplementary Figure 8I**). This remarkable phenotypic resilience translated directly into robust functional stability; expanded CD27^+^ AS-Tregs maintained their potent capacity to suppress both CD4^+^ and CD8^+^ effector T-cell proliferation, even following prolonged stimulation within this inflammatory microenvironment (**Figure 8D**).

Furthermore, comprehensive assessment of migratory markers following inflammatory stimulation revealed that the surface expression of CXCR3 (87.0 ± 11.0% vs. 82.1 ± 12.9% in controls), CCR4 (99.9 ± 0.3% vs. 99.9 ± 0.1%), CCR7 (43.0 ± 22.6% vs. 49.3 ± 14.1%), and CCR2 (54.7 ± 34.3% vs. 51.7 ± 34.7%) remained stable, with no significant differences between the cytokine-treated and standard control conditions (**Supplementary Figures 9A–D**). These findings were further corroborated by high-dimensional clustering, which demonstrated that exposure to inflammatory cytokines altered neither the overall chemokine receptor profile (**Supplementary Figure 9E**) nor the specific cluster composition of the expanded CD27^+^ AS-Tregs (**Figures 8E, F**; **Supplementary Figure 9F**).

**Figure 9:**
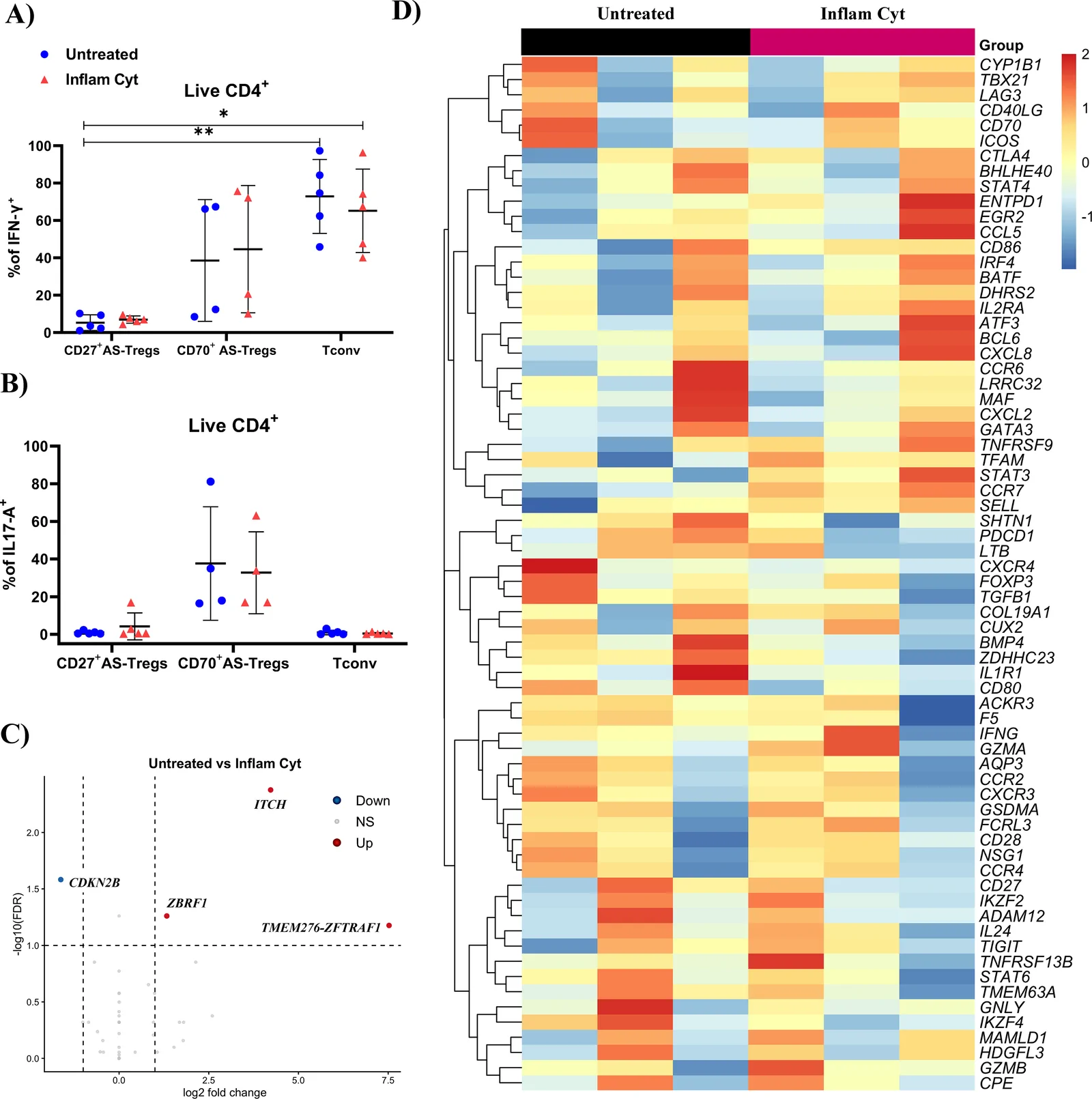
Expanded CD27^+^ AS-Tregs resist Th1/Th17 lineage conversion and maintain a stable regulatory transcriptome following inflammatory challenge. Expanded cells were cultured for an additional week in the presence of an inflammatory cytokine cocktail (IL-1β, IL-6, TNF-α, and IFN-γ) or standard control medium (Untreated). **(A, B)** Intracellular cytokine staining evaluating the potential conversion into pro-inflammatory effector lineages. Cumulative frequencies demonstrate that CD27^+^ AS-Tregs maintain negligible production of the Th1 cytokine IFN-γ **(A)** and the Th17 cytokine IL-17A **(B)**, even after prolonged inflammatory challenge. This robust resistance to pathogenic plasticity sharply contrasts with the elevated effector cytokine production observed in CD70^+^ AS-Tregs and Tconv cells. Data are presented as mean ± SD. Statistical analysis was performed using a Two-way ANOVA **(A, B)**. **(C)** Volcano plot illustrates the minimal differential gene expression between cytokine-challenged and untreated CD27^+^ AS-Tregs (n=4 per group). **(D)** Hierarchical clustering and heatmap of core regulatory and effector genes. The complete list of differentially expressed genes is provided in **Supplementary Tables 1–6**. * p < 0.05, ** p < 0.01.

Then, we assessed safety by determining if inflammatory stimulation induced trans-differentiation into effector lineages. CD27^+^ AS-Tregs exhibited negligible basal IFN-γ production (5.3 ± 4.1%) compared with Tconv (72.9 ± 19.7%; p < 0.01), and this low expression did not increase after one week of inflammatory cytokine stimulation (**Figure 9A**). Similarly, CD27^+^ AS-Tregs displayed minimal IL-17A expression (0.97 ± 0.8%) compared with CD70^+^ AS-Tregs (37.6 ± 30.2%), and this expression remained unchanged following inflammatory challenge (**Figure 9B**).

### 3.8 The transcriptomic profile of CD27^+^ AS-Tregs is preserved following inflammatory challenge

Finally, to determine whether exposure to inflammatory cytokines alters the transcriptional program of expanded CD27^+^ AS-Tregs, bulk RNA sequencing (RNA-seq) was performed. Volcano plot analysis revealed transcriptional similarity between untreated and inflammatory conditions, with only three genes differentially expressed (*ITCH*, *ZNRF1*, and *TMEM276*) (**Figure 9C**). Notably, key genes associated with Treg lineage identity and suppressive function—including *FOXP3*, *IL2RA*, *CTLA4*, *TIGIT*, *LRRC32*, *ENTPD1*, and *IKZF2*—remained stable, with no coordinated induction of pro-inflammatory or effector-associated transcripts. Heatmap analysis of a curated panel of immunologically relevant markers further supported these findings (**Figure 9D**), as samples from both conditions displayed comparable expression patterns and failed to segregate by treatment.

## 4 Discussion

In this study, we report that a selection strategy targeting alloantigen-specific CD25^+^CD27^+^CD70^−^ Tregs (CD27^+^ AS-Tregs) facilitates the generation of a stable regulatory population. To ensure genuine antigen specificity from the outset, cells were isolated following primary stimulation with allogeneic Mo-DCs, as cytokine supplementation alone was insufficient to induce proliferation (**Supplementary Figures 2A, B**). This primary stimulation phase included the addition of all-trans-retinoic acid (ATRA), which we previously determined to support optimal initial AS-Treg proliferation (19) and is known to promote the phenotypic stability of human Tregs (32, 33). Specifically, we isolated alloreactive AS-Tregs by sorting exclusively the actively dividing (CTV^low^) fraction (**Supplementary Figure 1**)—an approach associated with high enrichment for alloantigen-specific regulatory cells (34). To further optimize purity and long-term stability, our sorting strategy included the positive selection of CD27. This aligns with previous reports identifying CD27 as a useful marker to distinguish *bona fide* FOXP3^+^ Tregs from activated effector T cells, which can transiently upregulate CD25 upon TCR engagement (35, 36). Additionally, the concurrent exclusion of CD70^+^ cells was implemented to prevent the potential outgrowth of effector-like subsets during prolonged expansion. While the intrinsic regulatory properties of CD27 and CD70 on polyclonal Tregs are recognized (21), the novelty of our study lies in translating this biology into a combinatorial manufacturing pipeline. By integrating alloantigen-driven proliferation with targeted CD27 positive selection and CD70 exclusion, our strategy actively addresses the progressive epigenetic drift and lineage destabilization that frequently compromise standard, CD25-based long-term AS-Treg cultures (19).

The characterization of CD27^+^ AS-Tregs immediately after the 7-day co-culture revealed high expression of FOXP3, Helios, CTLA-4, and CD39, alongside minimal expression of the exhaustion marker TIM-3 (**Supplementary Figure 2C**). This profile is indicative of an initial regulatory, non-exhausted state (37). Importantly, the preservation of a high frequency of FOXP3^+^ cells within the CD27^+^ AS-Treg population after 21 days of culture is noteworthy, as clinical protocols require extended expansion to achieve target therapeutic doses. While our optimized culture cocktail—composed of IL-2, IL-15, TGF-β, and rapamycin—provides crucial signals for Treg fitness [with IL-2, IL-15, and TGF-β cooperatively maintaining the *FOXP3* locus open (2, 38, 39), and rapamycin suppressing Tconv cell outgrowth (40, 41)], the stable FOXP3 expression and sustained TSDR demethylation retained in our expanded CD27^+^ AS-Tregs likely reflect an intrinsic epigenetic feature of this specific subset (21). Although prolonged *ex vivo* proliferation inherently induces a degree of epigenetic drift compared to freshly isolated Tregs, the retention of approximately 60% demethylation represents a stable regulatory signature. Indeed, we previously demonstrated that similar rapamycin- and cytokine-based conditioning fails to maintain such stable *FOXP3*-TSDR profiles, leading to progressive remethylation, when AS-Tregs are isolated solely based on CD25 expression (19). This underscores that the initial sorting phenotype, rather than just the *in vitro* culture environment, is a critical determinant of long-term epigenetic stability and lineage maintenance.

Beyond lineage commitment, longitudinal tracking of per-cell protein density revealed stable MFI levels for FOXP3, CD27, and CD70 during the 21-day *ex vivo* expansion period (**Supplementary Figure 3**), indicating that the expression intensity of these key markers remains consistent throughout the manufacturing process. The sustained exclusion of CD70 expression is of biological relevance. As previously reported, CD70-expressing human Tregs can paradoxically provide costimulatory signals to conventional T cells, potentially impairing their overall *in vitro* regulatory capacity (21). Our phenotypic observations support the premise that this effector-like profile is stably maintained during culture in CD70-expressing subsets, thereby validating our rationale for physically excluding the CD70^+^ subpopulation during the initial cell sorting to optimize the purity and potential suppressive profile of the cell product.

To confirm that this phenotypic resilience is intrinsic rather than dependent on continuous pharmacological conditioning, we evaluated the phenotype of CD27^+^ AS-Treg following rapamycin withdrawal (**Supplementary Figure 7**). The core regulatory phenotype (FOXP3, CD25, Helios, CD27, CTLA-4) remained preserved after an additional week of culture without the drug, suggesting that the initial CD25^+^CD27^+^CD70^−^ isolation captures an imprinted regulatory program. Translationally, this drug-independent stability is advantageous, as clinical-grade manufacturing strictly requires the washout of culture compounds prior to patient infusion (5–8).

Beyond core lineage markers, high-dimensional analysis revealed that expanded CD27^+^ AS-Tregs display a distinct immunoregulatory signature characterized by the co-expression of Helios, CTLA-4, CD25, and CD39. The retention of Helios is critical, as it marks Tregs with enhanced immunosuppressive function and stability in inflammatory environments (42, 43), while high CTLA-4 levels are essential for the regulation of antigen-presenting cells and the maintenance of peripheral tolerance (44). Furthermore, the high expression of CD39 suggests these cells are equipped to modulate the metabolic microenvironment of the allograft via the adenosine pathway (45). In this context, a similar CD27^+^FOXP3^+^Helios^+^CTLA-4^+^CD39^+^ Treg phenotype has been recently identified as a driver of long-term xenograft tolerance in humanized mouse models (46), supporting the therapeutic potential of the CD27^+^ Tregs.

Additionally, the low levels of TIM-3 observed in long-term expanded CD27^+^ AS-Tregs likely reflects the ‘resting’ state at which the phenotype was evaluated, as TIM-3 is a TCR-inducible molecule primarily upregulated following acute activation (47). Since sustained TIM-3 expression is also associated with cellular exhaustion (37), its relative absence in our cells after extensive expansion suggests they have not reached terminal exhaustion.

Importantly, this stable molecular signature translated into a potent ability to suppress effector T cell proliferation *in vitro*. Expanded CD27^+^ AS-Tregs exhibited an enhanced anti-proliferative capacity compared to both standard polyclonal Tregs and the internal non-proliferating control (Non-AS Tregs), effectively controlling effector T cell (Teff) expansion even at demanding ratios. This divergence within the identical allogeneic co-culture demonstrates that heightened suppressive potency is intrinsically linked to alloantigen-driven proliferation. This observation aligns with Veerapathran et al., who validated that selecting the dividing Treg fraction yields a high suppressive alloreactive product (34). This profile is further supported by findings from Koenen et al., who demonstrated that unlike their CD27^−^ counterparts, only CD27^+^ Tregs can potently suppress ongoing alloimmune responses and memory T cell activation (48). Crucially, this suppression was strictly alloantigen-specific (**Figures 4C–F**), displaying robust inhibition of donor-directed responses while exhibiting minimal cross-suppression against third-party stimulation.

While these *in vitro* suppression assays validate the donor-reactive enrichment, we acknowledge certain limitations that warrant future investigation. First, the absence of direct clonal quantification remains a limitation; future high-throughput T cell receptor (TCR) sequencing will be essential to definitively characterize the clonality, diversity, and selective expansion of alloreactive clonotypes within this cell product (49, 50). Furthermore, while our data support the capacity of CD27^+^ AS-Tregs to suppress conventional T cell proliferation *in vitro*, we acknowledge that this represents only one aspect of Treg-mediated regulation. Future preclinical studies evaluating additional functional endpoints—such as their capacity to suppress the secretion of pro-inflammatory cytokines (e.g., IFN-γ, TNF-α) by effector T cells, or their ability to down-modulate costimulatory molecules on dendritic cells—will be essential to comprehensively define the complete regulatory arsenal and physiological efficacy of this therapeutic product.

A major concern in adoptive Treg therapy is the potential for effector cell overgrowth or the conversion of Tregs into non-regulatory phenotypes following prolonged *ex vivo* culture (17). Our RNA-seq data addresses this concern, demonstrating that expanded CD27^+^ AS-Tregs preserve a comprehensive Treg signature—characterized by high expression of *FOXP3*, *IKZF2* (Helios), *IKZF4* (Eos), *BATF*, *IL2RA* (CD25), *CD27*, *CTLA4*, *TIGIT*, *LRRC32* (GARP), *ICOS*, and *TNFRSF9* (CD137) (51, 52)—while actively repressing inflammatory programs. The coordinated expression of these specific genes suggests a functional and stable regulatory phenotype. Specifically, *IKZF4* (Eos) functions as an essential corepressor for FOXP3, securing lineage identity by silencing effector Th cell transcriptional programs (53–55). Simultaneously, the co-expression of *LRRC32* (GARP) and *ICOS* is critical for preventing lineage loss by sustaining FOXP3 expression—GARP via a positive feedback loop, and ICOS by promoting its epigenetic preservation—thereby securing a robust suppressive capacity (56, 57). Furthermore, the sustained expression of *BATF* suggests the potential for tissue accumulation, survival, and stability, properties that are associated with the control of inflammatory responses (58). Additionally, the upregulation of *TNFRSF9* (CD137) serves as a specific activation signature that identifies a stable Treg subset characterized by preserved epigenetic fidelity (59). This regulatory identity is further underscored by the heatmap segregation: while Tconv cells displayed a pro-inflammatory profile enriched for *IFNG*, *GZMA*, *GZMB*, and *CSF2* (GM-CSF), the CD27^+^ AS-Treg clusters lacked these cytolytic and Th-associated transcripts (51, 52).

In addition to suppression mechanisms, efficient Treg trafficking is a critical determinant for successful tolerance induction. In this study, we show that expanded CD27^+^ AS-Tregs exhibit a “dual-homing” phenotype, characterized by the simultaneous expression of lymphoid (CCR7/CCR2) and inflammatory (CXCR3, CCR4) receptors. The expression of CCR7 and CCR2 equips Tregs to recirculate through secondary lymphoid organs, a prerequisite for localizing within T-cell zones to access IL-2 and control early alloimmune priming (60–62). Simultaneously, the sustained expression of CCR4 and CXCR3 enables targeted migration into the inflamed allograft to suppress local effector responses—a process essential for maintaining long-term donor-specific tolerance (63, 64). Importantly, our functional assays confirmed that CXCR3 is biologically active, as CD27^+^ AS-Tregs efficiently migrated toward CXCL10. Collectively, these findings indicate that expanded CD27^+^ AS-Tregs possess the theoretical homing machinery required to access secondary lymphoid organs and inflamed tissues, although their *in vivo* migratory dynamics warrant further investigation.

Another major safety hurdle for adoptive cell therapy is the inflammatory microenvironment of transplant rejection, which can destabilize Tregs into pathogenic effector cells (65). Notably, even after prolonged exposure to a pro-inflammatory cytokine cocktail, our CD27^+^ AS-Tregs maintained their regulatory identity, chemokine receptor profile, and suppressive capacity, with negligible production of IFN-γ and IL-17A. This robust resistance to Th1/Th17 trans-differentiation is likely governed by two synergistic mechanisms. First, the deep demethylation of the *FOXP3*-TSDR acts as an epigenetic imprinting that safeguards lineage stability under inflammatory conditions (20). Second, as recently demonstrated by Cao et al., intrinsic CD27 signaling actively reinforces FOXP3 expression via the JAK3–STAT5 axis, preventing the cytokine-induced destabilization commonly observed in CD27^−^ Tregs (46).

Regarding transcriptomic resilience, the observation that only three genes were significantly differentially expressed following the inflammatory challenge is striking. While we acknowledge that the statistical power to detect low-magnitude transcriptomic shifts is formally limited by our sample size (n = 3 per group), the use of a high-throughput platform (Illumina NovaSeq X Plus) and a relatively permissive statistical threshold (FDR < 0.1) would likely capture large-scale lineage destabilization if it were present. Therefore, despite exposure to the inflammatory cocktail, CD27^+^ AS-Tregs globally maintained their core regulatory signature. Particularly relevant among the minimal changes detected was the significant upregulation of the E3 ubiquitin ligase ITCH under inflammatory conditions. This likely represents a protective adaptation, as ITCH acts as a critical checkpoint preventing Treg lineage instability and the acquisition of pathogenic Th2 properties (66). Furthermore, the complete absence of newly induced pro-inflammatory loci at the transcriptional level distinguishes our cells from unstable Treg subsets (65), highlighting their profound capacity to maintain a robust regulatory identity under inflammatory challenge.

A formal limitation of our study is the absence of an *in vivo* model. Although humanized mice are widely used to test human Treg efficacy (67–70), faithfully modeling human alloimmune responses is hindered by suboptimal Treg survival due to lacking cross-reactive cytokines, strong xenoreactivity, incomplete lymphoid organ development and the absence of human antigen-presenting cells (67, 70–76). Furthermore, the rapid onset of xenogeneic graft-versus-host disease (xGVHD) severely restricts the experimental window, meaning that observed immunosuppression often reflects the control of murine xenoantigens rather than strict human-to-human alloreactivity (67–69, 72, 75, 76). Technically, accurately distinguishing adoptively transferred Tregs from endogenous counterparts is challenging, frequently necessitating non-physiological doses that are difficult to extrapolate clinically (68, 76–78). Nevertheless, the translational potential of our findings aligns with recent preclinical studies demonstrating that CD27-enriched human Tregs exhibit prolonged graft accumulation and enhanced capacity to extend allograft survival compared to polyclonal populations (46, 79). These independent *in vivo* observations complement our phenotypic profiling, providing a rationale for evaluating this subset in subsequent specialized models.

On the other hand, recent advances in genetically engineered Tregs, particularly CAR- and TCR-Tregs, have introduced attractive approaches for inducing transplant tolerance. While offering distinct theoretical advantages—such as MHC-independent targeting for CAR-Tregs and tailored allorecognition for TCR-Tregs—these therapies currently face notable biological and translational hurdles. For instance, CAR-Tregs can be susceptible to tonic signaling-induced exhaustion and potential conversion toward inflammatory phenotypes, which may compromise their long-term *in vivo* persistence and suppressive function (37, 80). Similarly, the clinical application of TCR-Tregs can be complicated by TCR chain mispairing and a heightened reliance on exogenous IL-2 (81). In this context, unedited CD27^+^ AS-Tregs represent a translatable complementary strategy (46, 79). By expanding naturally occurring donor-reactive cells, this approach preserves a physiological TCR repertoire that may circumvent the exhaustion risks associated with synthetic receptors, harnessing their intrinsic stability to potentially favor long-term allograft acceptance (11, 12, 16).

In conclusion, we show that the selective expansion of CD25^+^CD27^+^CD70^−^ alloantigen-specific Tregs generates a stable cell product characterized by high proliferative capacity, robust epigenetic fidelity, and potent suppressive function *in vitro*. Notably, these cells exhibit a chemokine receptor profile consistent with dual-homing potential (lymph nodes and allograft) and firmly resist effector conversion under inflammatory challenge. While *in vivo* studies remain essential to fully validate their survival, homing dynamics, and capacity to induce long-term tolerance in a physiological context, our comprehensive findings position CD27^+^ AS-Tregs as promising candidates for a more stable Treg therapy in long term transplantation tolerance.

## Data Availability

The original contributions presented in the study are included in the article/**Supplementary Material**, further inquiries can be directed to the corresponding author/s.
